# A DPPH· Kinetic Approach on the Antioxidant Activity of Various Parts and Ripening Levels of Papaya (*Carica papaya* L.) Ethanolic Extracts

**DOI:** 10.3390/plants10081679

**Published:** 2021-08-15

**Authors:** Olimpia Alina Iordănescu, Maria Băla, Dina Gligor (Pane), Simelda Elena Zippenfening, Marius Ioan Cugerean, Mircea Ionuţ Petroman, Daniel Ioan Hădărugă, Nicoleta Gabriela Hădărugă, Mircea Riviş

**Affiliations:** 1 Department of Horticulture, Banat’s University of Agricultural Sciences and Veterinary Medicine “King Michael I of Romania” from Timişoara, Calea Aradului 119, 300645 Timişoara, Romania; olimpia.iordanescu@yahoo.com (O.A.I.); mariabalamonicabala@yahoo.com (M.B.); 2 Doctoral School “Engineering of Vegetable and Animal Resources”, Banat’s University of Agricultural Sciences and Veterinary Medicine “King Michael I of Romania” from Timişoara, Calea Aradului 119, 300645 Timişoara, Romania; gligor_dina_bihor@yahoo.com (D.G.); simelda_zippenfening@yahoo.com (S.E.Z.); marius_03cuge@yahoo.com (M.I.C.); daniel.hadaruga@upt.ro (D.I.H.); 3 SC Merpano SRL, Complexului Str. 10, Timiş County, 307370 Săcălaz, Romania; petromanionut@yahoo.com; 4 Department of Applied Chemistry, Organic and Natural Compounds Engineering, Polytechnic University of Timişoara, Carol Telbisz 6, 300001 Timişoara, Romania; 5 Department of Food Science, Banat’s University of Agricultural Sciences and Veterinary Medicine “King Michael I of Romania” from Timişoara, Calea Aradului 119, 300645 Timişoara, Romania; 6 Department of Dental Medicine, “Victor Babeş” University of Medicine and Pharmacy Timişoara, Eftimie Murgu Sq. 2, 300041 Timişoara, Romania; mrivis@yahoo.com

**Keywords:** unripe and ripe papaya, *Carica papaya* L., kinetics, antioxidant activity, radical scavenging capacity, DPPH· reaction rate, papaya extracts, non-alcoholic and alcoholic beverages

## Abstract

Papaya fruits (*Carica papaya* L.) are valuable both as food, including concentrates and mixed beverages and in traditional medicine. The goal of the study was to evaluate the antioxidant activity of various parts of unripe and ripe papaya fruit from the DPPH· kinetics point of view. Peel, pulp, seed, and seed-pulp of unripe and ripe papaya fruits (¼ and >¾ level of ripening) were extracted with ethanol and monitored at 517 nm in the presence of DPPH·. The radical scavenging capacity (*RSC*) at various time ranges and DPPH· reaction rates for specific time intervals were determined. The highest *RSC* values were obtained for papaya pulp extracts, consistently higher for the ripe samples in comparison with the unripe ones (86.4% and 41.3%). The DPPH· rates significantly differ for the unripe and ripe papaya extracts, especially for the first time range. They are more than double for the ripe papaya. These values were 2.70, 4.00, 3.25, 2.75 μM/s for the peel, pulp, seed, seed-pulp extracts from the ripe papaya and only 1.00, 1.65, 1.40, 1.80 μM/s for the unripe samples. DPPH· kinetic approach can be useful for a fast and simple evaluation of the overall antioxidant properties of fruit extracts.

## 1. Introduction

Papaya is the generic name of the tree and fruit of *Carica papaya* L., which belongs to the genus Carica, botanical family Caricaceae. It originates from Central and South America but is cultivated in all tropical regions. Papaya had various traditional and ethnic applications, both in the food and medicinal fields, such as culinary (cooked unripe fruit, raw ripe papaya, salads, savory dishes), non-alcoholic and alcoholic beverages (papaya concentrates, mixed beverages, papaya wine), food ingredients (papaya extracts for instant beverages, dietary fiber concentrates, glucoside-based isolates and seeds oil) as well as in the treatment of malaria, antimicrobial, purgative, anti-asthmatic, anti-diabetic, or the latex from unripe papaya against chronic skin ulcers, dermatitis and psoriasis due to the presence of specific endopeptidase proteolytic enzymes (papain, chymopapain, leukopapain, and caricain) [1,2,3,4,5,6,7,8,9]. By far, Asia is the main producer and exporter of papaya fruits (more than a half of the worldwide production), followed by Central and South America [5,10]. The fruit is harvested and commercialized at various ripening levels, such as green papaya (100% green shell) or papaya with ripening levels of <¼, ¼ to ½, ½ to ¾, and >¾ maturity levels corresponding to <25, 25–50, 50–75 and 75–100% yellow shell [1,5].

The composition of papaya fruit is different for unripe and ripe papaya, as well as for the various parts of the fruit. Nutritional compounds such as proteins, lipids, and fibers are more concentrated in the ripe fruits (up to 2%, 0.6%, and 1.7% in comparison with 1.1, 0.5, and 1.3% for the unripe fruit, respectively) [1,5]. No significant differences exist for the carbohydrate and water contents of unripe and ripe papaya (11–24 and 85–95%, respectively) [5,11]. Regarding the composition of the seed, the lipid fraction is the most concentrated (up to 60%), but soluble and insoluble proteins (up to 28%, mainly consisting of globulins—more than a half) and fibers (~23%) are also important [6,12].

Some of the most important biologically active compounds in papaya fruit are polyphenols. They are responsible for almost all pharmacological activities due to their antioxidant properties. Their composition varies during ripening (more concentrated in ripe fruits), fruit part, growing conditions (geographical region, sun exposure), storage, and processing of papaya fruits [6,10,13,14,15,16,17,18,19]. Among polyphenols (especially flavonoids and phenolic acids), carotenoids, saponins/triterpenoids, and ascorbic acid are also important for the overall antioxidant activity of papaya fruits. The main compounds from the flavonoid class found in papaya peel, pulp, and leaves were myricetin, quercetin, kaempferol, morin, apigenin, and luteolin. On the other hand, *p*-coumaric, caffeic, and ferulic acids were the main compounds from the hydroxycinnamic derivative class. Leaves also contain protocatechuic and chlorogenic acids, 5,7-dihydroxycoumarin, tannins, saponins, and proanthocyanidins, while papaya seeds especially contain phenolic tannins. Total phenolic compounds (expressed as mg gallic acid equivalents (GAE)/100 g) have values of 26-75.6 mg GAE/100 g and up to 1263 mg GAE/100 g for fresh and dry papaya pulp, respectively [5,16,17,20,21]. On the other hand, this parameter had lower values in the papaya peel and leaves. On the contrary, the total flavonoid content was higher for papaya leaves than for fresh pulp (up to 17 mg catechin equivalents (CE)/100 g in comparison with 3 mg CE/100 g, respectively) [17]. The dried fresh pulp can contain up to 65 mg quercetin equivalents/100 g [5,16]. Moreover, tannins were found in fresh papaya pulp at similar contents such as for flavonoids (1.25–3.36 mg CE/100 g fresh sample), while seeds have a tannin concentration of 6.35 mg/100 g dry sample [4,5,6,12,22,23]. Other compounds having antioxidant activities that were identified in papaya fruit belong to the anthocyanin class. Their total contents, expressed as cyanidin-3-*O*-glucoside (C-3-G)/100 g sample, were only ~1.9 mg C-3-G/100 g dry papaya pulp [5]. Regarding the particular compounds having antioxidant activity, they were quantified in various parts of papaya fruit by liquid chromatographic (HPLC—high-performance liquid chromatography, coupled with diode array detector, DAD, or mass spectrometry detector, MS) and spectrophotometric techniques [1,3,7,14,16,22,24]. Among flavonoids, myricetin (3 mg/100 g), as well as morin, quercetin, and kaempferol (2 mg/100 g for every compound) were identified in Hawaiian fresh papaya using HPLC/UV-Vis and HPLC/DAD techniques [25]. It was observed that the concentration of hydroxycinnamic acids decreases during ripening, from 230 to 136 mg *p*-coumaric acid/100 g, from 176 to 113 mg caffeic acid/100 g, and from 277 to 187 mg ferulic acid/100 g dry papaya peel, as determined by HPLC-DAD [26]. Caffeic and ferulic acids were also identified in papaya fruit (46–68 and 133–162 mg/100 g dry pulp), as well as rutin from flavonoid glycoside class (100–160 mg/100 g dry pulp) [27]. Carotenoids such as β-cryptoxanthin and lycopene, as well as ascorbic acid were also quantified in papaya fruit at levels of 0.31–0.80, 0.15–1.2/1.7, and 63–73.7/25.1–58.6 mg/100 g dry sample (Hawaiian/Mexican), respectively [26,27].

Chemical and enzyme-based methods are used for the evaluation of the antioxidant activity of compounds and extracts [7,21,28]. Mostly, chemical methods were used for the analysis of the antioxidant properties of papaya. Among these, DPPH· (2,2-diphenyl-1-pycrylhydrazyl), FRAP (ferric reducing antioxidant power), ABTS^+^ (2,2′-azino-bis(3-ethylbenzthiazoline-6-sulfonic acid, radical cation), ORAC (oxygen radical absorbance capacity) methods, TEAC (Trolox equivalent antioxidant capacity), with 6-hydroxy-2,5,7,8-tetramethylchroman-2-carboxylic acid as standard, and Folin-Ciocalteu technique for total phenols were generally applied [1,2,6,7,14,16,17,18,19,20,21,22,25,26,29]. Dietary fiber concentrates from papaya pulp and peel revealed antioxidant capacity, as determined by DPPH· and FRAP methods [1]. Soluble and hydrolyzable polyphenols isolated from organic and conventional papaya cultivars (pulp and peel) were important for the overall antioxidant activity. It was observed that the samples obtained from organic papaya have higher antioxidant activity, according to DPPH· evaluation [20]. There are many studies on the antioxidant activity of papaya pulp [17,21,24,26], seed [6,23], leaf [18,19,29] or carotenoid-enriched peel extracts [14,15,16,25,26], using various chemical methods. However, there are some studies regarding the antioxidant activity of papaya seed extracts using enzyme-based methods such as superoxide dismutase (SOD), catalase (CAT), and glutathione peroxidase (GPx) as endogenous antioxidant enzymes [10,23].

There are some studies regarding the evaluation of DPPH· reaction kinetics, especially for pure compounds such as *tert*-butylhydroxytoluene, *tert*-butylhydroxyanisole, *tert*-butylhydroquinone, ascorbyl palmitate, tocopherol, rutin, eugenol, isoeugenol, gallic and ascorbic acids [30,31,32,33,34,35], or fruit and plant extracts having antioxidant properties (lemon and pomegranate juices, green tea infusion, rosemary extract and essential oil, olive and grape seed extracts) [33,35]. Up to now, no kinetic studies related to papaya antioxidant properties were performed.

The main goal of the study was to emphasize the DPPH· kinetics on the discrimination between papaya ethanolic extracts, especially regarding the level of ripening. This approach was for the first time performed for such extracts and can be useful as a fast and simple evaluation method of the overall antioxidant properties of fruit extracts.

## 2. Results and Discussion

### 2.1. Radical Scavenging Capacity of Unripe and Ripe Papaya Extracts

Papaya fruit contains various compounds having antioxidant properties, which were widely studied (e.g., ascorbic acid, myricetin from flavonoid class, or cinnamic acid derivatives) [1,5,24,25,26,27]. The present study was only focused on the overall antioxidant activity of ethanolic extracts obtained from unripe and ripe papaya parts, using the DPPH· method. The free radical DPPH· has a maximum absorbance at 517 nm, where no significant absorbance appears for other antioxidant compounds (or other biologically active compounds) from fruit extracts [33,36,37,38]. Consequently, the monitoring of the absorbance of the mixture of DPPH· solution and papaya extract allows evaluating the *RSC* of the last through the reaction of DPPH· radical with the antioxidant compounds from the extract. The absorbance of the reaction products (neutral DPPH-H and degradation compounds resulted from the antioxidant compounds from the extract) is not significant at the measuring wavelength (517 nm). They present absorbance at lower wavelength values than 450 nm, according to previous studies [36,37,38]. As a result of the consumption of DPPH· during the interaction with the papaya extract, the absorbance of the mixture at 517 nm linearly decreases with the increase of the *RSC*, according to Equation (1) (see Section 3.3).

Representative *RSC* values for papaya extracts were obtained at the beginning of the antioxidant activity determination (after 5 s of DPPH·—antioxidant compound reaction), as well as at 1, 5, and 15 min of reaction (Table 1, Figures S1 and S2). The *RSC versus Time* plot differs for the papaya peel extracts in comparison with pulp, seed, and seed-pulp extracts, for both unripe and ripe cases (see also Tables S1–S4 for the *p*-values). The *RSC* values have a slower increase for the peel extracts. This behavior can be observed by comparison of the *RSC* values at the beginning and the end of the antioxidant activity evaluation. They are more than sixteen times higher at 15 min in comparison with values at 5 s in the case of peel extract from the unripe papaya (code *Lu*). For comparison, the *RSC* value for the peel extract from the ripe papaya (code *Lr*) is almost two times higher at the end of the analysis. In all other cases, *RSC* values are less than 1.3 times higher at the end of the analysis, in comparison with the beginning of antioxidant activity determination (1.17–1.37 and 1.02–1.26 times higher for unripe and ripe cases, respectively). However, the overall *RSC* data after 15 min of analysis reveals the highest values for all extracts from the ripe papaya parts, especially for the pulp extract (86.44 and 68.1% for the ripe and unripe papaya pulp extracts, respectively; *p* < 0.02). The differences are also statistically significant for the other papaya extracts (Table S4): 68.10 and 39.95% for the ripe and unripe papaya peel extracts (*p* < 0.075), 74.76 and 32.76% for the ripe and unripe papaya seed extracts (*p* < 0.05), 60.66 and 41.05% for the ripe and unripe papaya seed-pulp extracts (*p* < 0.10), respectively. These differences are more obvious at the intermediate time intervals, such as at 5 s (*p* < 0.04 for all cases, especially for the ripe and unripe papaya pulp extracts, *p* < 0.005, Table S1), as well as at 1 min (*p* < 0.09, Table S2).

**Figure 1:**
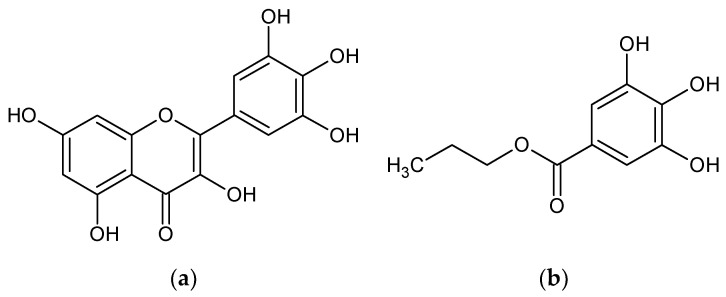
Comparison between chemical structures of myricetin from papaya fruit (**a**) and the standard compound propyl gallate (**b**).

These *RSC* values were compared with those obtained for standard compounds such as propyl gallate (PG), caffeic acid (CA), or *tert*-butylated hydroxyanisole (BHA). Ethanolic solutions of standard compounds at various concentrations were subjected to the same DPPH· technique and *RCS* values at the same time ranges were recorded (according to the work of Ivanovici et al. [37] and Table S5). The *RSC* values for the ripe papaya pulp extracts are similar to those obtained for PG solution at a concentration of 0.2 mM (*RSC* of 83.32% at 15 min, Table S5). On the other hand, the corresponding extract obtained from unripe papaya pulp has similar *RSC* values to the 0.1 mM PG solution (*RSC* of 44.86%). Moreover, the extracts obtained from the unripe papaya pulp from the seeds region (code *SPu*, *RSC* of 41.05%) have similar antioxidant activity behavior such as the same 0.1 mM PG solution. This behavior suggests the involvement of similar antioxidant structures in the DPPH· radical scavenging (i.e., myricetin from papaya and the standard compound propyl gallate, Figure 1).

The monitoring of the antioxidant activity was also performed for other fruit extracts such as pomegranate or kiwi [36,38]. The highest *RSC* values in the case of pomegranate extracts were obtained for red and white peel ethanolic extracts (48.94–64.99% after 15 min; Table S6), while for kiwi fruit the best results were obtained for the peel extracts obtained using 60% ethanol solution (84.1% after 15 min; Table S7). Galang et al. [2] evaluated the antioxidant activity of the methanol-water fractionated extracts obtained from unripe *C. papaya* L. and compared with ascorbic acid as the standard antioxidant compound. The *RSC* values after 30 min were almost as high as for the standard compound in the case of the last fractions (five fractions of a total of twelve having *RSC* values of 91–96%, in comparison with ascorbic acid, with an *RSC* value of 98%). An increase of the antioxidant activity with the ripening of papaya fruit was also observed [7,39]. The antioxidant activity of papaya seeds extracts was evaluated for various extraction techniques, i.e., subcritical water extraction (SWE) and conventional Soxhlet technique [22]. The antioxidant activity was expressed as the DPPH· concentration required to reduce the absorbance by 50%, EC_50_. It was observed that the increase of extraction temperature in SWE from 70 to 150 °C determines the decrease of the EC_50_ values from 4.1 to 1.67 μg/mL, while the conventional technique provided an intermediate value of 3.74 μg/mL. Higher values for the DPPH· inhibitory concentration 50% of papaya seeds methanol extracts (1.0 mg/mL) were obtained by Salla et al. [23]. In another study performed by Zhou et al. [40], the best results on antioxidant activity of papaya seed extracts were obtained if more hydrophobic solvents were used. Thus, ethyl acetate provided the best results and some phenolic compounds such as *p*-hydroxybenzoic and vanillic acids were isolated and identified in these extracts (as antioxidant compounds among already known ones, such as tocopherols). In conclusion to these comparisons, the present study reveals similar antioxidant activities with some of the above-mentioned extracts. Thus, ripe papaya peel and pomegranate white peel extracts have similar antioxidant activity behavior. The same observation can be done for the ripe papaya pulp and kiwi peel extracts. This behavior suggests the presence of the same type of antioxidants in these extracts, which react with DPPH· radical.

Further, the monitoring of the antioxidant activity through the DPPH· kinetics of radical scavenging of papaya extracts was studied.

### 2.2. DPPH· Reaction Kinetics in the Presence of the Unripe and Ripe Papaya Extracts

**Figure 2:**
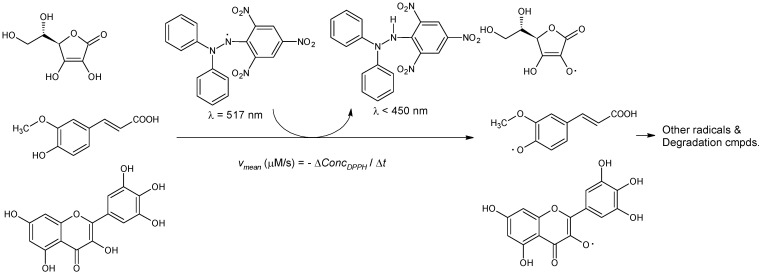
Schematic radical reaction between DPPH· and antioxidant compounds from papaya fruit extracts (ascorbic acid, ferulic acid, and myricetin).

The reaction of the stable radical DPPH· with various antioxidant compounds from the papaya extracts is presented in Figure 2. Antioxidant compounds such as ascorbic acid, ferulic acid, and myricetin react with DPPH· in order to generate the neutral compound DPPH-H (2,2-diphenyl-1-pycrylhydrazine) and other radical intermediates, which are further transformed in neutral degradation compounds [41,42]. Only DPPH· radical has significant absorbance at 517 nm, while the neutral compounds do not absorb in this region. The maximum absorbance of DPPH· is shifted to a much lower value after these reactions (<450 nm [41,42]). On the other hand, papaya extracts do not have significant absorbance at 517 nm (e.g., ascorbic acid has a maximum absorbance in the UV region at 243 nm, ferulic acid at 291 and 321 nm, while myricetin has a maximum absorbance at 328 nm [43,44,45]). Consequently, the monitoring of the absorbance at 517 nm for the DPPH· and papaya extract mixture allows to determine the actual DPPH· concentration. Considering the variation of DPPH· concentration in a specific time range, the overall mean DPPH· reaction rate can be calculated (Equations (2) and (3); see Section 3.4).

**Figure 3:**
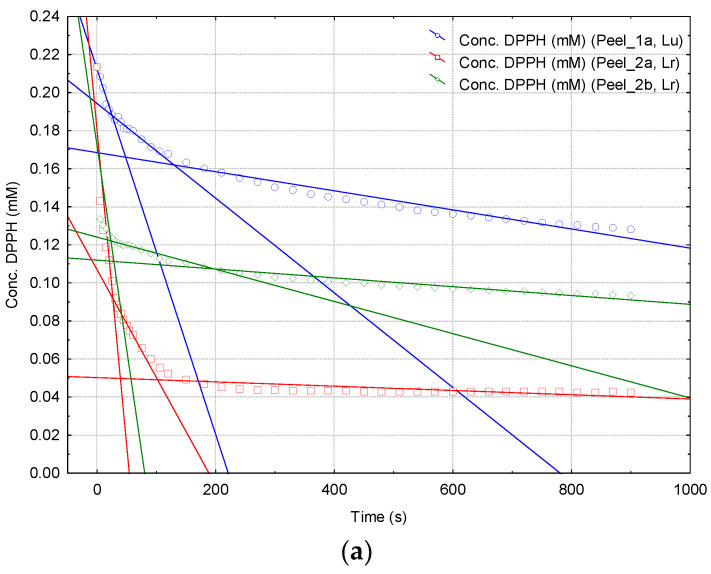

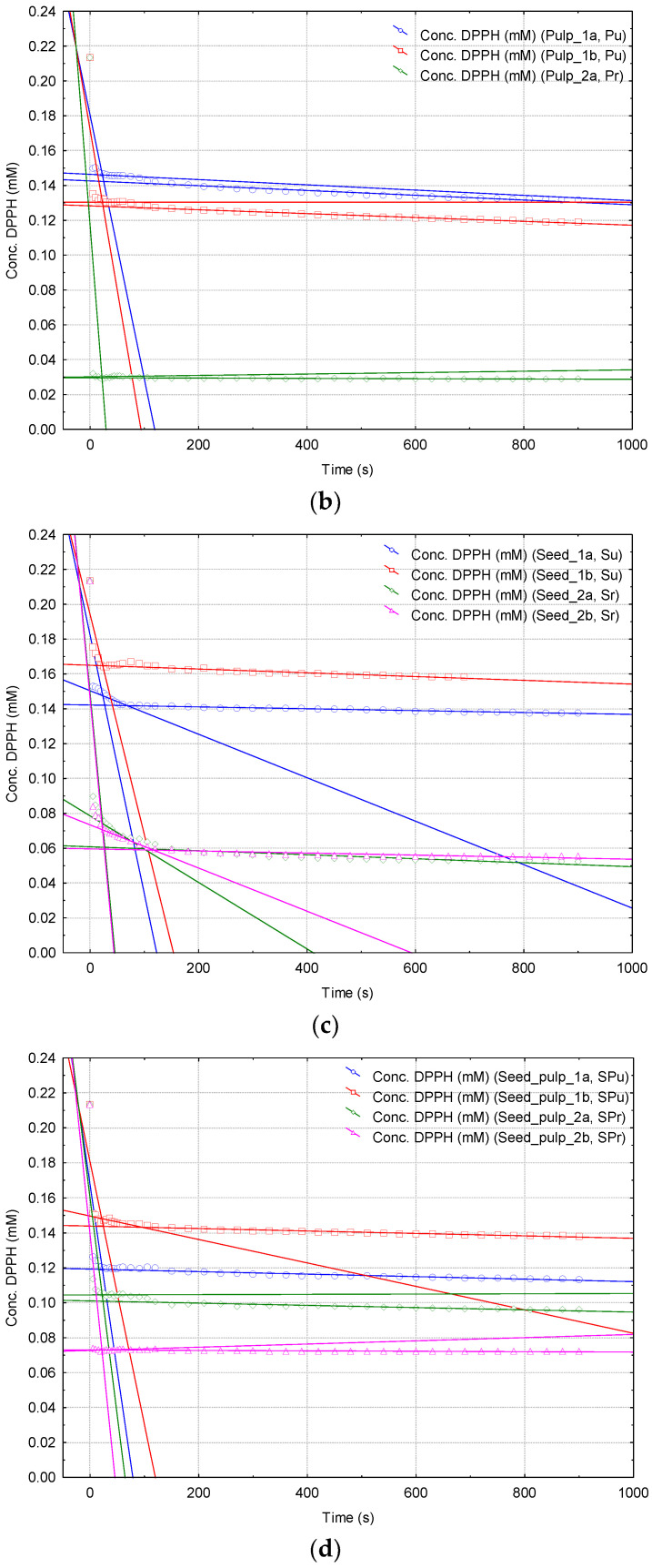
*Concentration* (DPPH·, mM) *versus Time* (s) plots for the reaction of DPPH· with antioxidant compounds from unripe and ripe papaya peel extracts (codes *Lu* and *Lr*) (**a**), from the unripe and ripe papaya pulp extracts (codes *Pu* and *Pr*) (**b**), from the unripe and ripe papaya seeds extracts (codes *Su* and *Sr*) (**c**) and from the unripe and ripe papaya seed-pulp extracts (codes *SPu* and *SPr*) (**d**). Determinations were performed as duplicates (excepting *Lu* and *Pr* samples). See Supplementary Material File for all results.

Generally, the reaction of DPPH· with the antioxidant compounds from papaya fruit extracts exhibits a decreasing of the absorbance at 517 nm that has three specific time ranges: 0–30 s, where the antioxidant compounds with higher reactivity interact with DPPH· (e.g., ascorbic acid), 30–80 s and 80–900 s, corresponding to DPPH· reaction with antioxidant compounds having lower reactivity (e.g., compounds with hindered hydroxyl groups such as in the case of ferulic acid or flavonoids, as well as the synthetic standard compound, BHA). These regions are also revealed in the *Conc.* (DPPH·) *versus Time* plots (Figures S3–S10). Correlations on these regions provide the mean variation of DPPH· concentration (decrease) on the specific time range, which means the overall mean DPPH· reaction rates (*v_mean(1–3)_*, Equation (3), Figure 3). All DPPH· reaction rates, expressed as μM/s, are presented in Table 2.

There are significant differences between the DPPH· reaction rate values in the first time range. Thus, *v_1_* is 1.53–2.70 times higher in the case of the ripe papaya extracts, in comparison with the corresponding unripe fruit extracts, the highest difference being obtained for the peel extracts (2.70 μM/s for the ripe papaya peel extract and only 1.00 μM/s for the unripe case, Table 2). Slightly lower differences were also obtained for pulp and seed extracts (4.00 μM/s and 3.25 μM/s for the ripe papaya extracts, 1.65 μM/s and 1.40 μM/s for the corresponding unripe fruit extracts, respectively; Table 2). However, the absolute DPPH· reaction rates are higher in these last cases. Differences between DPPH· reaction rate values for the unripe and ripe papaya extracts are statistically significant in all cases. *P*-values were lower than 0.043 (except for *SPu* and *SPr* cases, *p* < 0.098), with the lowest value for the papaya pulp extracts (*p* < 0.016; see Tables S8–S10 in Supplementary Material File for all *p*-values). On the contrary, DPPH· reaction rates for the last two time ranges were not relevant for the differentiation between the unripe and ripe papaya extracts. The mean reaction rate in the second time range was five to twelve times lower (<0.34 μM/s) than the corresponding *v_1_* values, while *v_3_* was much lower (values of 0.001-0.050 μM/s). Kinetic results on the papaya fruit extracts are in agreement with the kinetic data for both standard antioxidant compounds and other fruit extracts [36,37,38]. Regarding the standard antioxidant compounds such as PG, CA, and BHA, the DPPH· reaction rates on similar time ranges were in the same region. Thus, the highest *v_1_* values were obtained for CA and PG solutions at concentrations of 1 mM (2.50 and 1.90 μM/s, but for the time range of 0-60 s, respectively; Table S11), which are close to the *v_1_* values for the ripe papaya extracts. On the other hand, unripe papaya extracts have similar kinetic behavior with the standard compounds PG, CA, and BHA at concentrations of 0.1, 0.2/0.1, and 1.0 mM, respectively (1.20–1.30 μM/s, Table S11). Higher *v_1_* values, which are close to the corresponding DPPH· reaction rate values for the ripe papaya extracts, were also obtained for other fruit extracts, such as pomegranate red and white peel and pulp extracts (2.43–3.03 μM/s for the time range of 0–30 s, Table S12) [38].

## 3. Materials and Methods

### 3.1. Materials

**Figure 4:**
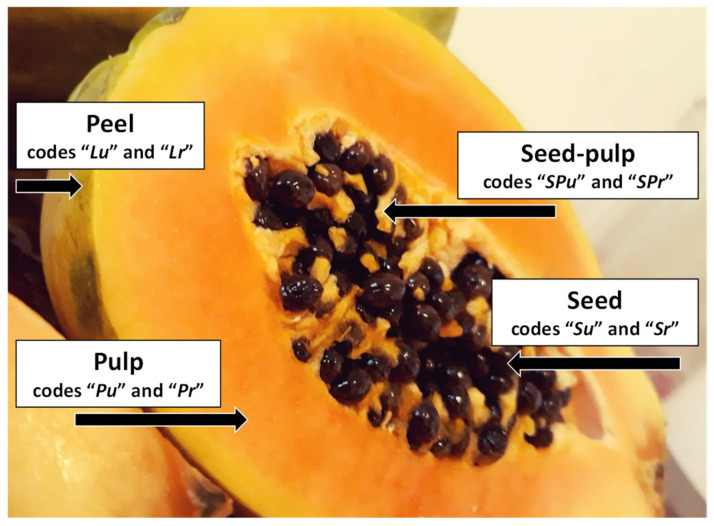
Representative image for the papaya parts used in this study: peel (the exterior of the fruit, codes “*Lu*” and “*Lr*” for the unripe and ripe papaya), pulp (the main core part, without the seeds region, codes “*Pu*” and “*Pr*” for the unripe and ripe fruit), seed (without the surrounding pulp, codes “*Su*” and “*Sr*” for the unripe and ripe fruit) and seed-pulp (the pulp from the seeds region, codes “*SPu*” and “*SPr*” for the unripe and ripe papaya).

Papaya (*Carica papaya* L.) fruits were purchased from the local market (Timişoara, Romania) in the autumn of 2019. Fruits were imported from South America and two types of papaya fruits were selected, according to the ripening level: unripe papaya fruit with ¼ level of ripening and ripe papaya fruit with >¾ level of ripening (Figure 4). The level of ripening was evaluated according to the works of Calvache et al. and Ikram et al. [1,5], the unripe samples having a maturity stage of “2” (25–50% yellow surface, surrounded by light green), while the ripe papaya had a maturity stage of “4” (>75% yellow surface). The fresh samples (two fruits for every type of papaya) were manually separated into four parts: peel (the exterior of the fruit, codes “*Lu*” and “*Lr*” for the unripe and ripe papaya), pulp (the main core part, without the seeds region, codes “*Pu*” and “*Pr*” for the unripe and ripe fruit), seed (without the surrounding pulp, codes “*Su*” and “*Sr*” for the unripe and ripe fruit) and seed-pulp (the pulp from the seeds region, codes “*SPu*” and “*SPr*” for the unripe and ripe papaya). Papaya samples were stored at 4 °C until extraction. A representative image for these parts of papaya fruit used in this study is presented in Figure 4.

Other reagents were ethanol 96% (volumetric concentration, *pro analysis* grade, Chimopar, Bucharest) and 2,2-diphenyl-1-pycrylhydrazyl (DPPH·, >99%, Merck & Co., Inc., Kenilworth, NJ, USA).

### 3.2. Obtaining Papaya Fruit Extracts

The extracts of papaya fruit were obtained by solid-liquid extraction using mild conditions for protecting antioxidant compounds against degradation. Thus, the fresh samples were well ground in a mortar at room temperature and 5.0 g were weighed for every extraction. The extraction was performed in a 150 mL sealed flask in the presence of 20 mL ethanol 96% by intermittent agitation at 25 (±1) °C for 120 h. The extract was then filtered and the solid residue was washed with ethanol. The filtrate was diluted with ethanol at a final volume of 25 mL for the antioxidant activity evaluation and kinetic studies. Extracts were obtained as duplicates from two different fruits for every type of papaya, unripe and ripe (four parts: peel, pulp, seeds, and pulp-seeds). All extracts were made in parallel: 2 × 4 extracts for unripe papaya (two unripe fruits, maturity stage of “2”) and 2 × 4 extracts for ripe papaya (two ripe fruits, maturity stage of “4”). For some cases, spectrophotometric monitoring cannot be performed up to the end of measurements (i.e., sample duplicates of *Lu* and *Pr* cannot be included in the evaluation; see Table 1).

### 3.3. Evaluation of the Antioxidant Activity by DPPH· Method

The antioxidant activity was evaluated through the radical scavenging capacity (*RSC*) of the papaya extracts in the presence of DPPH· [36,37,38]. The absorbance of a mixture of 0.5 mL papaya extract, 0.5 mL 0.1 mM DPPH· ethanolic solution, and 2 mL ethanol was monitored for 900 s at 517 nm in a 10 mm length quartz cuvette, using a CamSpec M501 single-beam Scanning UV-Visible spectrophotometer (CamSpec Ltd., Cambridge, United Kingdom). The “Time Scan” module was used. Actual *RSC_t_* values were obtained using Equation (1), where *A_t_* and *A_0_* stand for the absorbance of the papaya extract and DPPH· solution at the reaction time *t* and *t* = 0, respectively (Equation (1)).

*RSC_t_* (%) = [1 − *A_t_*/*A_0_*]·100 (1)

### 3.4. Evaluation of the DPPH· Reaction Rates

The actual DPPH· concentration was determined by means of a calibration curve obtained for DPPH· solutions in the range of 0–300 μM. The following DPPH· calibration curve was obtained (Equation (2)).

*Conc.* (DPPH·, μM) = 159.58(±2.80)·*Absorbance(@517 nm)* (2)

*n* = 5, *r^2^* = 0.999, *F* = 3244, *s* = 6.64, *p* < 10^−6^.

**Figure 5:**
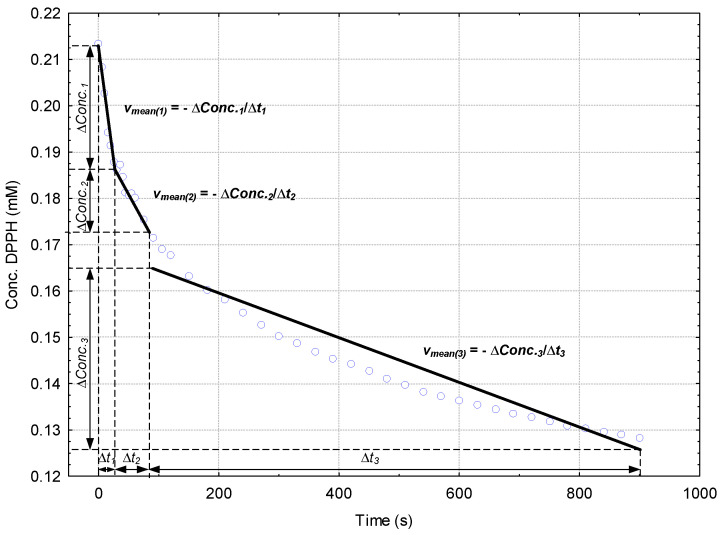
Schematic representation of the calculus of the mean DPPH· reaction rates (*v_mean(1–3)_*) from the linear correlations of the *Concentration* (DPPH·) *versus Time* plots for the specific time ranges: 0–30 s (Δ*t_1_*), 30–80 s (Δ*t_2_*) and 80–900 s (Δ*t_3_*).

For papaya extracts, three pseudo-linear time ranges in the *Concentration* (DPPH·) *versus Time* plot can be observed. In the first time range of 0–30 s a fast reaction of antioxidant compounds with DPPH· appears. In the second and third time ranges of 30–80 s and 80–900 s, the reaction rates are consistently lower (see Figure 5 and Supplementary Material File). Thus, the mean DPPH· reaction rates for these three time ranges (*v_mean(1–3)_*, μM/s) were determined according to the following equation (Equation (3)), where Δ*Conc.* stands for the variation of the DPPH· concentration (μM) during the time range Δ*t* (s), for the above-mentioned time ranges (“*1*” for 0–30 s, “*2*” for 30–80 s and “*3*” for 80–900 s), (Equation (3)).

*v_mean(1−3)_* (μM/s) = −Δ*Conc._DPPH (1−3)_* (μM)/Δ*t_(1−3)_* (s) (3)

### 3.5. Statistical Analysis

*RSC* and DPPH· reaction rate values are expressed as mean (±standard deviation, SD). Correlations between dependent and independent variables were performed by linear regression analysis (DPPH· concentration and reaction rate as dependent variables, as well as absorbance or time as independent variables). Linear regression analysis also provides standard error values for regression coefficients, standard error of estimate (*s*), correlation and determination coefficients (*r^2^* and *r^2^_adjusted_*), Fisher (*F*), and *p*-values. Significant differences of the values for the *RSC* of unripe and ripe papaya, various parts, were obtained by Fisher LSD (least significant difference) test. The parametrization was sigma-restricted, while the confidence limit and significance level were set at 0.95 and 0.05, respectively. The *one-way ANOVA* and *Multiple Linear Regression* modules in Statistica 7.1 software (StatSoft, Inc., Tulsa, OK, USA) were used for statistical analysis.

## 4. Conclusions

A DPPH· kinetic approach for discriminating between the unripe and ripe papaya (*Carica papaya* L.) fruit ethanolic extracts was proposed for the first time. The radical scavenging capacity clearly differs for the ripe and unripe papaya extracts for all studied fruit parts, i.e., peel, pulp, seed, and pulp from the seed region. By far, the highest antioxidant activity was observed for the ripe papaya pulp extract, which is 1.2–1.4 times higher than the other ripe papaya extracts at the end of antioxidant activity monitoring. Comparing these radical scavenging capacity values for the ripe and unripe papaya extracts, the values for the first cases are consistently higher (1.5–2.3 times higher for the ripe cases, with the highest difference for the extracts of the seeds). The DPPH· reaction rates are significantly different in the case of interaction with the antioxidant compounds from the ripe and unripe papaya extracts for the beginning of the reaction. The highest overall mean DPPH· reaction rates were obtained in the case of the ripe papaya pulp extracts. They can be compared with the DPPH· reaction behavior of standard antioxidant compounds resembling those occurring in the papaya fruit (according to other studies; the quantification of specific antioxidant compounds was not the goal of the present study). These similarities were obtained for gallate and hydroxycinnamic acid moieties, which resembles the antioxidant flavonoids (e.g., myricetin) and caffeic/ferulic acids found in papaya fruit. In conclusion, kinetics approach on the first time range of DPPH· reaction can be useful for a fast and simple evaluation of the overall antioxidant properties of fruit extracts designed for food, pharmaceutical or cosmetic applications (such as non-alcoholic and alcoholic beverages, food supplements, antimicrobial, anti-inflammatory or antioxidant products, anti-aging, anti-acne, and natural skincare products) as well as for the differentiation and evaluation of the level of ripening of papaya used for obtaining the hydrophilic extracts.

## Figures and Tables

**Table 1:** Antioxidant activity results (*RSC*—radical scavenging capacity, %, at various reaction times) for papaya extracts (unripe and ripe, various parts; peel—*Lu* and *Lr*, pulp—*Pu* and *Pr*, seed—*Su* and *Sr*, seed-pulp—*SPu* and *SPr*). Values are expressed as mean (±standard deviation, SD), excepting * for single analysis. For a given parameter (*RSC*), values with different superscript letters are significantly different, according to Fisher LSD (least significant difference) test (*p* < 0.05). All *p*-level values are presented in the Supplementary Material File.

N^o^	Code	*RSC* (5 s) (%)	*RSC* (1 min) (%)	*RSC* (5 min) (%)	*RSC* (15 min) (%)
1	Lu *	2.43 ^a^	15.57 ^a^	29.66 ^a^	39.95 ^a^
2	*Lr*	35.21 (±3.17) ^b^	55.12 (±15.40) ^b^	65.66 (±19.65) ^b^	68.10 (±16.88) ^b^
3	*Pu*	33.16 (±4.76) ^b^	35.21 (±4.87) ^c^	38.56 (±4.30) ^a^	41.31 (±4.17) ^a^
4	Pr *	84.97 ^c^	85.74 ^d^	86.23 ^bc^	86.44 ^bc^
5	*Su*	23.96 (±8.80) ^bd^	29.00 (±9.51) ^e^	31.39 (±9.67) ^a^	32.76 (±9.96) ^a^
6	*Sr*	59.31 (±1.84) ^e^	68.98 (±0.25) ^bf^	73.63 (±0.36) ^b^	74.76 (±0.94) ^b^
7	*SPu*	35.06 (±8.23) ^b^	37.71 (±8.14) ^c^	39.61 (±8.26) ^a^	41.05 (±8.23) ^a^
8	*SPr*	56.08 (±13.21) ^e^	58.17 (±10.39) ^b^	60.01 (±8.53) ^b^	60.66 (±7.91) ^b^

**Table 2:** DPPH· reaction rates (*v_1–3_*—μM/s, at *t_1–3_* time ranges: 0–30 s, 30–80 s, and 80–900 s, respectively) for papaya extracts (unripe and ripe, various parts; peel—*Lu* and *Lr*, pulp—*Pu* and *Pr*, seed—*Su* and *Sr*, seed-pulp—*SPu* and *SPr*). Values are expressed as mean (±standard deviation, SD), excepting * for single value. For a given parameter (*v_1–3_*), values with different superscript letters are significantly different, according to Fisher LSD (least significant difference) test (*p* < 0.05, excepting *SPu* and *SPr* cases, with *p* < 0.10). All *p*-level values are presented in the Supplementary Material File.

No	Code	DPPH· Reaction Rate on *t_1_* Time Range, *v_1_* (μM/s)	DPPH· Reaction Rate on *t_2_* Time Range, *v_2_* (μM/s)	DPPH· Reaction Rate on *t_3_* Time Range, *v_3_* (μM/s)
1	Lu *	1.00 * ^a^	0.20 *	0.050 *^a^
2	*Lr*	2.70 (±0.85) ^b^	0.34 *	0.017 (±0.008) ^b^
3	*Pu*	1.65 (±0.21) ^a^	-	0.013 (±0.002) ^b^
4	Pr *	4.00 *^bc^	-	0.001 *^c^
5	*Su*	1.40 (±0.14) ^a^	0.10 *	0.008 (±0.004) ^b^
6	*Sr*	3.25 (±0.07) ^b^	0.15 (±0.07)	0.009 (±0.004) ^b^
7	*SPu*	1.80 (±0.42) ^a^	0.07 *	0.007 *^b^
8	*SPr*	2.75 (±0.35) ^b^	-	0.004 *^bc^

## Data Availability

Not applicable.

## Supplementary Materials

The following are available online at www.mdpi.com/article/10.3390/plants10081679/s1, Figure S1: The variation of *RSC* (%) *versus Time* (s) for the extracts obtained from unripe papaya parts in the presence of DPPH· ethanolic solution: (a) unripe papaya extract from peel (one determination); (b) unripe papaya extract from pulp; (c) unripe papaya extract from seeds; (d) unripe papaya extract from seed-pulp (surrounding pulp from the seeds region), Figure S2: The variation of *RSC* (%) *versus Time* (s) for the extracts obtained from ripe papaya parts in the presence of DPPH· ethanolic solution: (a) ripe papaya extract from peel; (b) ripe papaya extract from pulp (one determination); (c) ripe papaya extract from seeds; (d) ripe papaya extract from seed-pulp (surrounding pulp from the seeds region), Table S1: Significant *p*-levels from the Fisher’s LSD test (least significant difference) for the antioxidant activity of papaya extracts (*RSC*—radical scavenging activity, %, at 5 s from the initiation of DPPH· reaction; unripe papaya peel, pulp, seed and seed-pulp: *Lu*, *Pu*, *Su* and *SPu*; ripe papaya peel, pulp, seed and seed-pulp: *Lr*, *Pr*, *Sr* and *SPr*). *p*-level values lower than 0.05 are bolded, Table S2: Significant *p*-levels from the Fisher’s LSD test (least significant difference) for the antioxidant activity of papaya extracts (*RSC*—radical scavenging activity, %, at 1 min from the initiation of DPPH· reaction; unripe papaya peel, pulp, seed, and seed-pulp: *Lu*, *Pu*, *Su*, and *SPu*; ripe papaya peel, pulp, seed, and seed-pulp: *Lr*, *Pr*, *Sr*, and *SPr*). *p*-level values lower than 0.05 are bolded, Table S3: Significant *p*-levels from the Fisher’s LSD test (least significant difference) for the antioxidant activity of papaya extracts (*RSC*—radical scavenging activity, %, at 5 min from the initiation of DPPH· reaction; unripe papaya peel, pulp, seed, and seed-pulp: *Lu*, *Pu*, *Su,* and *SPu*; ripe papaya peel, pulp, seed, and seed-pulp: *Lr*, *Pr*, *Sr,* and *SPr*). *p*-level values lower than 0.05 are bolded, Table S4: Significant *p*-levels from the Fisher’s LSD test (least significant difference) for the antioxidant activity of papaya extracts (*RSC*—radical scavenging activity, %, at 15 min from the initiation of DPPH· reaction; unripe papaya peel, pulp, seed, and seed-pulp: *Lu*, *Pu*, *Su,* and *SPu*; ripe papaya peel, pulp, seed, and seed-pulp: *Lr*, *Pr*, *Sr,* and *SPr*). *p*-level values lower than 0.05 are bolded, Table S5: Antioxidant activity results (*RSC*—radical scavenging capacity, %, at various reaction times) for standard antioxidant compounds (*PG_1mM/0.2mM/0.1mM/0.05mM*—propyl gallate ethanolic solutions at concentrations of 1, 0.2, 0.1 and 0.05 mM; *CA_1mM/0.2mM/0.1mM/0.05mM*—caffeic acid ethanolic solutions at concentrations of 1, 0.2, 0.1 and 0.05 mM; *BHA_1mM*—*tert*-butylated hydroxyanisole ethanolic solution at a concentration of 1 mM). Values are means of duplicate analysis, with *RSD* < 5%, Table S6: Antioxidant activity results (*RSC*—radical scavenging activity, %, at various reaction times) for other fruit extracts (*Pg_J(1:50/1:100)*—pomegranate raw juice, 1:50 and 1:100 dilution; *Pg_Rp/Wp(1:50/1:100)*—pomegranate red/white peel ethanolic extracts, 1:50 and 1:100 dilution; *Pg_P/S*—pomegranate pulp and seed ethanolic extracts, 1:50 and 1:100 dilution). Values are expressed as mean (±standard deviation, SD) for multiplicate analysis (*n* = 7 for *Pg_J* samples and *n* = 3 for *Pg_Rp*, *Pg_Wp*, *Pg_P,* and *Pg_S* samples), Table S7: Antioxidant activity results (*RSC*—radical scavenging activity, %, at various reaction times) for other fruit extracts (*Kw_P20/60/100*—kiwi pulp extracts in 20%, 60%, and absolute ethanol; *Kw_L20/60/100*—kiwi peel extracts in 20%, 60%, and absolute ethanol). Values are means of duplicate analysis, with *RSD* < 5%, Figure S3: *DPPH· concentration* (mM) *versus Time* (s) for the unripe papaya peel extract (code *Lu*), one determination, Figure S4: *DPPH· concentration* (mM) *versus Time* (s) for the ripe papaya peel extract (code *Lr*), duplicate analysis, Figure S5: *DPPH· concentration* (mM) *versus Time* (s) for the unripe papaya pulp extract (code *Pu*), duplicate analysis, Figure S6: *DPPH· concentration* (mM) *versus Time* (s) for the ripe papaya pulp extract (code *Pr*), one determination, Figure S7: *DPPH· concentration* (mM) *versus Time* (s) for the unripe papaya seed extract (code *Su*), duplicate analysis, Figure S8: *DPPH· concentration* (mM) *versus Time* (s) for the ripe papaya seed extract (code *Sr*), duplicate analysis, Figure S9: *DPPH· concentration* (mM) *versus Time* (s) for the unripe papaya seed-pulp extract (code *SPu*), duplicate analysis, Figure S10: *DPPH· concentration* (mM) *versus Time* (s) for the ripe papaya seed-pulp extract (code *SPr*), duplicate analysis, Table S8: Significant *p*-levels from the Fisher’s LSD test (least significant difference) for the DPPH· reaction rate on the *t_1_ = 0–30 s* time range in the presence of papaya extracts (*v_1_*, μM/s; unripe papaya peel, pulp, seed and seed-pulp: *Lu*, *Pu*, *Su* and *SPu*; ripe papaya peel, pulp, seed and seed-pulp: *Lr*, *Pr*, *Sr* and *SPr*). *p*-level values lower than 0.05 are bolded, Table S9: Significant *p*-levels from the Fisher’s LSD test (least significant difference) for the DPPH· reaction rate on the *t_2_* = *30–80 s* time range in the presence of papaya extracts (*v_2_*, μM/s; unripe papaya peel, pulp, seed, and seed-pulp: *Lu*, *Pu*, *Su,* and *SPu*; ripe papaya peel, pulp, seed, and seed-pulp: *Lr*, *Pr*, *Sr,* and *SPr*). *p*-level values lower than 0.05 are bolded, Table S10: Significant *p*-levels from the Fisher’s LSD test (least significant difference) for the DPPH· reaction rate on the *t_3_* = *80–900 s* time range in the presence of papaya extracts (*v_3_*, μM/s; unripe papaya peel, pulp, seed, and seed-pulp: *Lu*, *Pu*, *Su,* and *SPu*; ripe papaya peel, pulp, seed, and seed-pulp: *Lr*, *Pr*, *Sr,* and *SPr*). *p*-level values lower than 0.05 are bolded, Table S11: DPPH· reaction rates (*v_1–3_*—μM/s, at *t_1-3_* time ranges: 0–60 s, 60–180 s, and 180–900 s, respectively) for standard antioxidant compounds (*PG_1mM/0.2mM/0.1mM/0.05mM*—propyl gallate ethanolic solutions at concentrations of 1, 0.2, 0.1 and 0.05 mM; *CA_1mM/0.2mM/0.1mM/0.05mM*—caffeic acid ethanolic solutions at concentrations of 1, 0.2, 0.1 and 0.05 mM; *BHA_1mM/0.2mM/0.1mM/0.05mM*—*tert*-butylated hydroxyanisole ethanolic solutions at concentrations of 1, 0.2, 0.1 and 0.05 mM). Values are means of duplicate analysis, with *RSD* < 5%, Table S12: DPPH· reaction rates (*v_1–3_*—μM/s, at *t_1-3_* time ranges: 0–30 s, 30–90 s, and 90–900 s, respectively) for other fruit extracts (*Pg_J(1:50/1:100)*—pomegranate raw juice, 1:50 and 1:100 dilution; *Pg_Rp/Wp(1:50/1:100)*—pomegranate red/white peel ethanolic extracts, 1:50 and 1:100 dilution; *Pg_P/S*—pomegranate pulp and seed ethanolic extracts, 1:50 and 1:100 dilution). Values are expressed as mean (±standard deviation, SD) for multiplicate analysis (*n* = 7 for *Pg_J* samples and *n* = 3 for *Pg_Rp*, *Pg_Wp*, *Pg_P,* and *Pg_S* samples).
