# Supplementary material for: A DPPH· Kinetic Approach on the Antioxidant Activity of Various Parts and Ripening Levels of Papaya (Carica papaya L.) Ethanolic Extracts
Source: Plants (Basel). 2021 Aug 15;10(8):1679. doi: 10.3390/plants10081679 (PMC8398966; doi:10.3390/plants10081679)
Supplement: Supplementary file 1 [file plants-10-01679-s001.zip › plants-1343778-supplementary.pdf]

plants

MDPI

Type of the Paper (Article)

Supplementary material

A DPPH· Kinetic Approach on the Antioxidant Activity of Various Parts and Ripening Levels of Papaya (*Carica papaya* L.) Ethanolic Extracts

Olimpia Alina Iordănescu [1,¶], Maria Băla [1,¶], Dina Gligor (Pane) [2], Simelda Elena Zippenfening [2], Marius Ioan Cugerean [2], Mircea Ionuţ Petroman [3], Daniel I. Hădărugă [2,4], Nicoleta G. Hădărugă [2,5,*], Mircea Riviş [6]

[1] Department of Horticulture, Banat's University of Agricultural Sciences and Veterinary Medicine "King Michael I of Romania" from Timişoara, Calea Aradului 119, 300645-Timişoara, Romania; olimpia.iordanescu@yahoo.com (O.A.I.); mariabalamonicabala@yahoo.com (M.B.)

[2] Doctoral School "Engineering of Vegetable and Animal Resources", Banat's University of Agricultural Sciences and Veterinary Medicine "King Michael I of Romania" from Timişoara, Calea Aradului 119, 300645-Timişoara, Romania; gligor_dina_bihor@yahoo.com (D.G.P.); simelda_zippenfening@yahoo.com (S.E.Z.); marius_03cuge@yahoo.com (M.I.C)

[3] SC Merpano SRL, Complexului Str. 10, 307370 – Săcălaz, Timiş county, Romania; petromanionut@yahoo.com (M.I.P.)

[4] Department of Applied Chemistry, Organic and Natural Compounds Engineering, Polytechnic University of Timişoara, Carol Telbisz 6, 300001 – Timişoara, Romania; daniel.hadaruga@upt.ro (D.I.H.)

[5] Department of Food Science, Banat's University of Agricultural Sciences and Veterinary Medicine "King Michael I of Romania" from Timişoara, Calea Aradului 119, 300645 –Timişoara, Romania; nicoletahadaruga@usab-tm.ro; nico_hadaruga@yahoo.com (N.G.H.)

[6] Department of Dental Medicine, "Victor Babeş" University of Medicine and Pharmacy Timişoara, Eftimie Murgu Sq. 2, 300041-Timişoara, Romania, mrivis@yahoo.com (M.R.)

* Correspondence: nico_hadaruga@yahoo.com; Tel.: +40-256-277-423

¶ These authors contributed equally to this work. They are both principal authors.

Citation: Iordănescu, O.A.; Băla, M.; Gligor (Pane), D.; Zippenfening, S.E.; Cugerean, M.I.; Petroman, M.I.; Hădărugă, D.I.; Hădărugă, N.G.; Riviş, M. A DPPH· Kinetic Approach on the Antioxidant Activity of Various Parts and Ripening Levels of Papaya (*Carica papaya* L.) Ethanolic Extracts. *Plants* **2021**, *10*, 1679. https://doi.org/10.3390/plants10081679

Publisher's Note: MDPI stays neutral with regard to jurisdictional claims in published maps and institu-tional affiliations.

Copyright: © 2021 by the authors. Licensee MDPI, Basel, Switzerland. This article is an open access article distributed under the terms and conditions of the Creative Commons Attribution (CC BY) license (http://creativecommons.org/licenses/by/4.0/).

Abstract: Papaya fruits (*Carica papaya* L.) are valuable both as food, including concentrates and mixed beverages, and in traditional medicine. The goal of the study was to evaluate the antioxidant activity of various parts of unripe and ripe papaya fruit from the DPPH· kinetics point of view. Peel, pulp, seed and seed-pulp of unripe and ripe papaya fruits (¼ and >¾ level of ripening) were extracted with ethanol and monitored at 517 nm in the presence of DPPH·. The radical scavenging capacity (*RSC*) at various time ranges and DPPH· reaction rates for specific time intervals were determined. The highest *RSC* values were obtained for papaya pulp extracts, consistently higher for the ripe samples in comparison with the unripe ones (86.4% and 41.3%). The DPPH· rates significantly differ for the unripe and ripe papaya extracts, especially for the first time range. They are more than double for the ripe papaya. These values were 2.70, 4.00, 3.25, 2.75 μM/s for the peel, pulp, seed, seed-pulp extracts from the ripe papaya and only 1.00, 1.65, 1.40, 1.80 μM/s for the unripe samples. DPPH· kinetic approach can be useful for a fast and simple evaluation of the overall antioxidant properties of fruit extracts.

Keywords: unripe and ripe papaya; *Carica papaya* L.; kinetics; antioxidant activity; radical scavenging capacity; DPPH· reaction rate; papaya extracts; non-alcoholic and alcoholic beverages

1. Radical scavenging capacity of unripe and ripe papaya

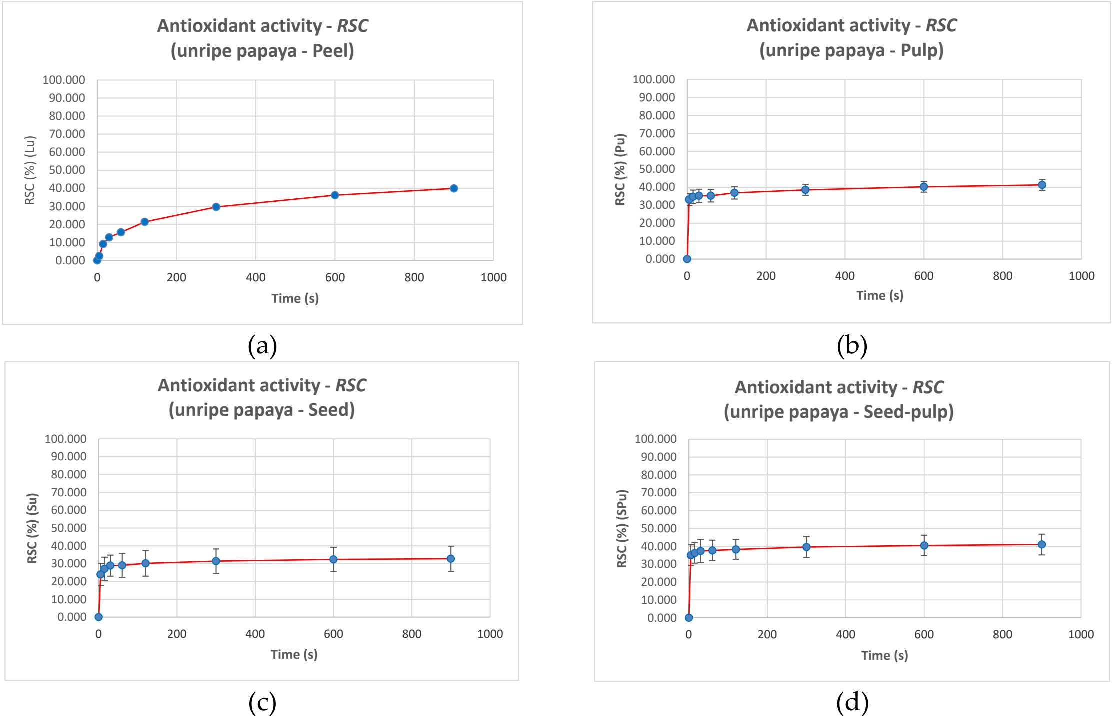

Figure S1. The variation of *RSC* (%) *versus Time* (s) for the extracts obtained from unripe papaya parts in the presence of DPPH· ethanolic solution: (a) unripe papaya extract from peel (one determination); (b) unripe papaya extract from pulp; (c) unripe papaya extract from seeds; (d) unripe papaya extract from seed-pulp (surrounding pulp from the seeds region).

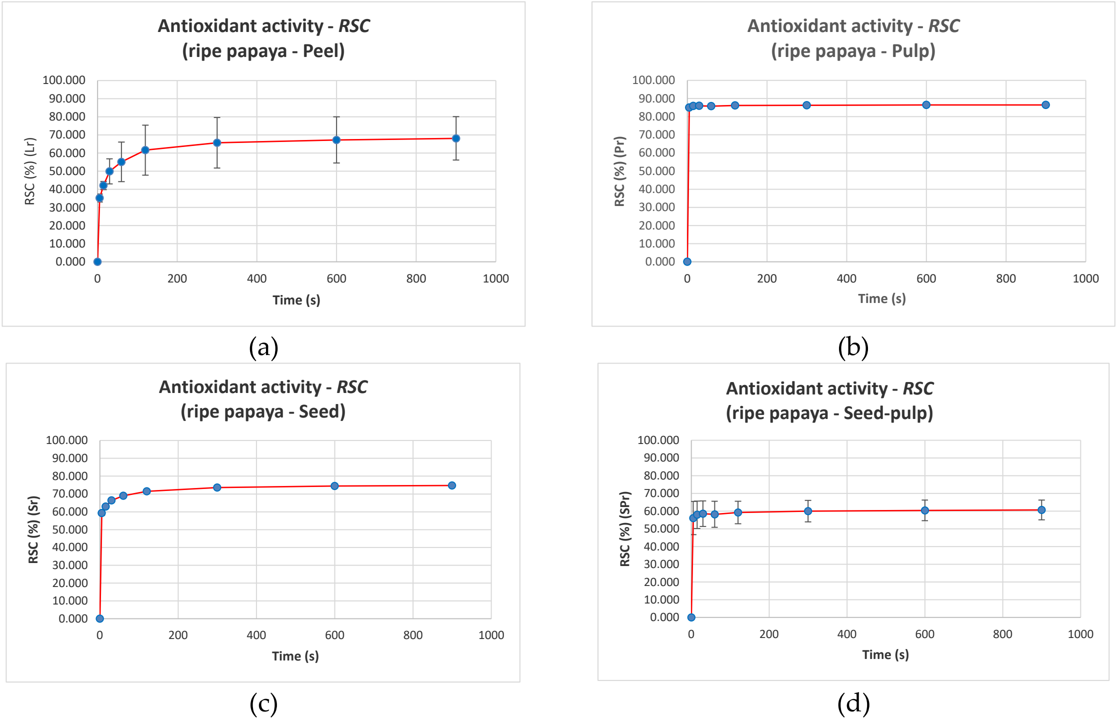

Figure S2. The variation of *RSC* (%) *versus Time* (s) for the extracts obtained from ripe papaya parts in the presence of DPPH· ethanolic solution: (a) ripe papaya extract from peel; (b) ripe papaya extract from pulp (one determination); (c) ripe papaya extract from seeds; (d) ripe papaya extract from seed-pulp (surrounding pulp from the seeds region).

Table S1. Significant *p*-levels from the Fisher's LSD test (least significant difference) for the antioxidant activity of papaya extracts (*RSC* – radical scavenging activity, %, at 5 s from the initiation of DPPH· reaction; unripe papaya peel, pulp, seed and seed-pulp: *Lu*, *Pu*, *Su* and *SPu*; ripe papaya peel, pulp, seed and seed-pulp: *Lr*, *Pr*, *Sr* and *SPr*;). *p*-level values lower than 0.05.

	Lu	*Lr*	*Pu*	*Pr*	*Su*	*Sr*	*SPu*	*SPr*
Lu		**0.021042**	**0.025855**	**0.001296**	0.053859	**0.003058**	0.059249	**0.003793**
Lr			0.791882	**0.004989**	0.601529	**0.029300**	0.538517	**0.045126**
Pu				**0.004309**	0.753851	**0.022658**	0.681674	**0.034198**
Pr					**0.005922**	**0.044662**	**0.005571**	**0.031333**
Su						**0.030521**	0.931513	**0.043432**
Sr							**0.027660**	0.679093
SPu								**0.039114**
SPr								

Table S2. Significant *p*-levels from the Fisher's LSD test (least significant difference) for the antioxidant activity of papaya extracts (*RSC* – radical scavenging activity, %, at 1 min from the initiation of DPPH· reaction; unripe papaya peel, pulp, seed and seed-pulp: *Lu*, *Pu*, *Su* and *SPu*; ripe papaya peel, pulp, seed and seed-pulp: *Lr*, *Pr*, *Sr* and *SPr*;). *p*-level values lower than 0.05 are bolded.

	Lu	*Lr*	*Pu*	*Pr*	*Su*	*Sr*	*SPu*	*SPr*
Lu		**0.028284**	0.170572	**0.006676**	0.212305	**0.010504**	0.294668	**0.022345**
Lr			0.106890	0.059857	0.174544	0.222450	0.120233	0.766243
Pu				**0.012691**	0.967248	**0.024539**	0.795432	0.075085
Pr					**0.021164**	0.227368	**0.016678**	0.079100
Su						**0.047485**	0.794925	0.128958
Sr							**0.034584**	0.323542
SPu								0.089675
SPr								

Table S3. Significant *p*-levels from the Fisher's LSD test (least significant difference) for the antioxidant activity of papaya extracts (*RSC* – radical scavenging activity, %, at 5 min from the initiation of DPPH· reaction; unripe papaya peel, pulp, seed and seed-pulp: *Lu*, *Pu*, *Su* and *SPu*; ripe papaya peel, pulp, seed and seed-pulp: *Lr*, *Pr*, *Sr* and *SPr*;). *p*-level values lower than 0.05 are bolded.

	Lu	*Lr*	*Pu*	*Pr*	*Su*	*Sr*	*SPu*	*SPr*
Lu		0.054618	0.541644	**0.021549**	0.608266	**0.030311**	0.802662	0.085867
Lr			0.068263	0.199009	0.109757	0.505909	0.075793	0.632393
Pu				**0.023549**	0.981152	**0.032615**	0.738403	0.121243
Pr					**0.036000**	0.399771	**0.027412**	0.121612
Su						0.057255	0.787432	0.179004
Sr							**0.040806**	0.280449
SPu								0.121526
SPr								

Table S4. Significant *p*-levels from the Fisher's LSD test (least significant difference) for the antioxidant activity of papaya extracts (*RSC* – radical scavenging activity, %, at 15 min from the initiation of DPPH· reaction; unripe papaya peel, pulp, seed and seed-pulp: *Lu*, *Pu*, *Su* and *SPu*; ripe papaya peel, pulp, seed and seed-pulp: *Lr*, *Pr*, *Sr* and *SPr*;). *p*-level values lower than 0.05 are bolded.

	Lu	***Lr***	***Pu***	***Pr***	***Su***	***Sr***	***SPu***	***SPr***
Lu		0.074085	0.913431	**0.026359**	0.991059	**0.041067**	0.744239	0.151771
Lr			**0.048724**	0.192485	0.072989	0.524585	**0.048460**	0.480014
Pu				**0.018251**	0.903203	**0.024940**	0.630847	0.113015
Pr					**0.026073**	0.375131	**0.019320**	0.092559
Su						**0.040512**	0.752553	0.149355
Sr							**0.027898**	0.214413
SPu								0.095611
SPr								

Table S5. Antioxidant activity results (*RSC* – radical scavenging capacity, %, at various reaction times) for standard antioxidant compounds (*PG_1mM/0.2mM/0.1mM/0.05mM* – propyl gallate ethanolic solutions at concentrations of 1, 0.2, 0.1 and 0.05 mM; *CA_1mM/0.2mM/0.1mM/0.05mM* – caffeic acid ethanolic solutions at concentrations of 1, 0.2, 0.1 and 0.05 mM; *BHA_1mM* – *tert*-butylated hydroxyanisole ethanolic solution at a concentration of 1 mM). Values are means of duplicate analysis, with *RSD* < 5% (from [1], with permission).

**N°**	**Code**	***RSC (1 min)*** **(%)**	***RSC (5 min)*** **(%)**	***RSC (15 min)*** **(%)**
1	*PG_1mM*	93.72	93.77	93.98
2	*PG_0.2mM*	69.52	77.89	83.32
3	*PG_0.1mM*	39.50	41.91	44.86
4	*PG_0.05mM*	27.99	24.79	26.27
5	*CA_1mM*	65.55	90.30	92.41
6	*CA_0.2mM*	32.49	48.82	53.08
7	*CA_0.1mM*	37.12	31.67	32.95
8	*CA_0.05mM*	13.16	14.37	14.81
9	*BHA_1mM*	22.19	68.21	86.46

Table S6. Antioxidant activity results (*RSC* – radical scavenging activity, %, at various reaction times) for other fruit extracts (*Pg_J(1:50/1:100)* – pomegranate raw juice, 1:50 and 1:100 dilution; *Pg_Rp/Wp(1:50/1:100)* – pomegranate red/white peel ethanolic extracts, 1:50 and 1:100 dilution; *Pg_P/S* – pomegranate pulp and seed ethanolic extracts, 1:50 and 1:100 dilution). Values are expressed as mean (±standard deviation, SD) for multiplicate analysis ($n = 7$ for *Pg_J* samples and $n = 3$ for *Pg_Rp*, *Pg_Wp*, *Pg_P* and *Pg_S* samples) (from [2], with permission).

Nº	Code	*RSC (1.5 min)* (%)	*RSC (15 min)* (%)
1	*Pg_J(1:50)*	15.99 (±3.22)	33.55 (±5.36)
2	*Pg_J(1:100)*	8.03 (±2.76)	17.68 (±5.11)
3	*Pg_Rp(1:50)*	46.97 (±5.64)	48.94 (±5.43)
4	*Pg_Rp(1:100)*	54.19 (±5.75)	58.38 (±7.96)
5	*Pg_Wp(1:50)*	46.88 (±4.44)	48.39 (±4.54)
6	*Pg_Wp(1:100)*	54.87 (±7.62)	64.99 (±6.22)
7	*Pg_P(1:50)*	50.9 (±2.58)	53.3 (±2.97)
8	*Pg_P(1:100)*	48.51 (±6.81)	59.32 (±5.23)
9	*Pg_S(1:50)*	5.12 (±0.34)	9.48 (±0.32)
10	*Pg_S(1:100)*	3.91 (±0.91)	7.22 (±1.77)

Table S7. Antioxidant activity results (*RSC* – radical scavenging activity, %, at various reaction times) for other fruit extracts (*Kw_P20/60/100* – kiwi pulp extracts in 20%, 60% and absolute ethanol; *Kw_L20/60/100* – kiwi peel extracts in 20%, 60% and absolute ethanol). Values are means of duplicate analysis, with *RSD* < 5% (from [3], with permission).

Nº	Code	*RSC (15 min)* (%)
1	*Kw_P20*	24.50
2	*Kw_P60*	14.80
3	*Kw_P100*	41.00
4	*Kw_L20*	49.30
5	*Kw_L60*	84.10
6	*Kw_L100*	55.80

2. DPPH· reaction kinetics in the presence of the unripe and ripe papaya extracts

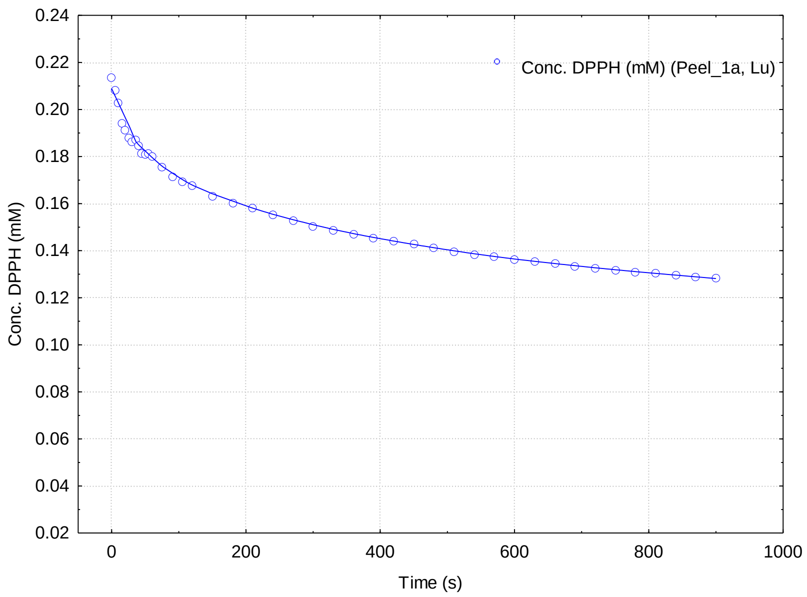

Figure S3. *DPPH· concentration* (mM) *versus Time* (s) for the unripe papaya peel extract (code *Lu*), one determination.

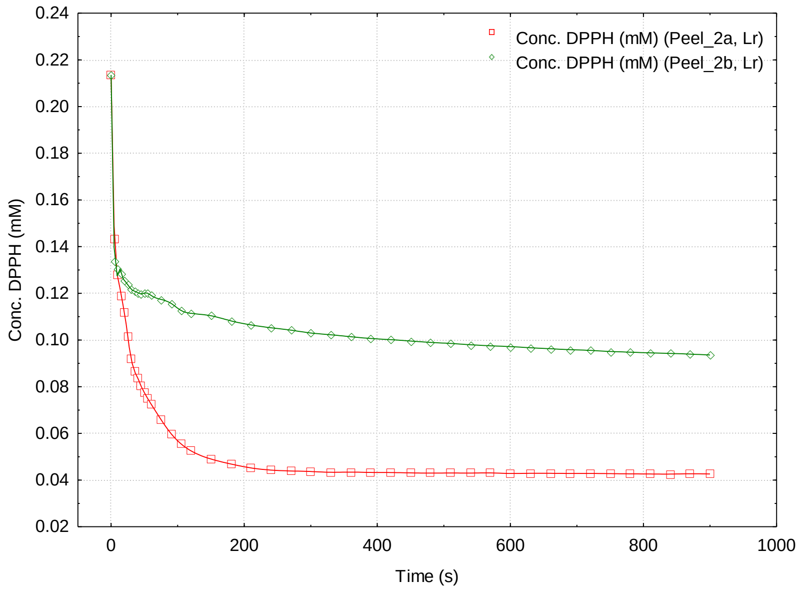

Figure S4. *DPPH· concentration* (mM) *versus Time* (s) for the ripe papaya peel extract (code *Lr*), duplicate analysis.

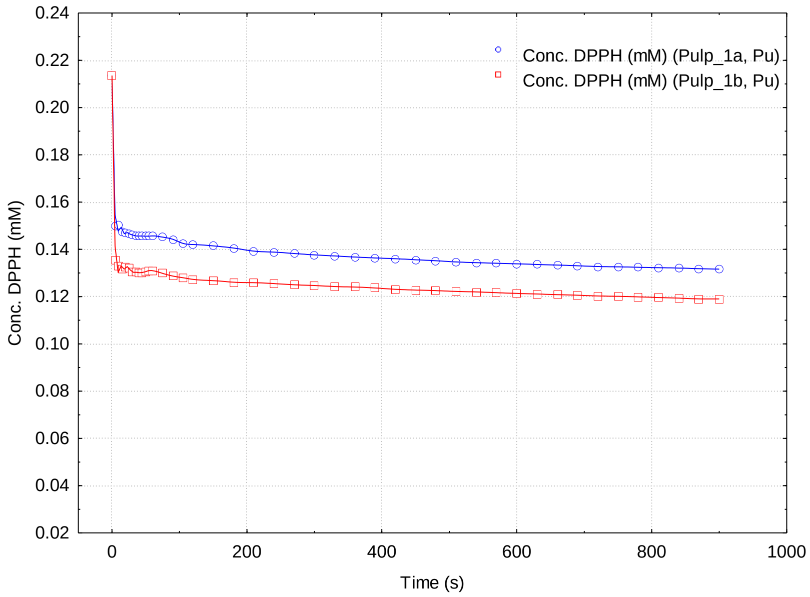

Figure S5. *DPPH· concentration* (mM) *versus Time* (s) for the unripe papaya pulp extract (code *Pu*), duplicate analysis.

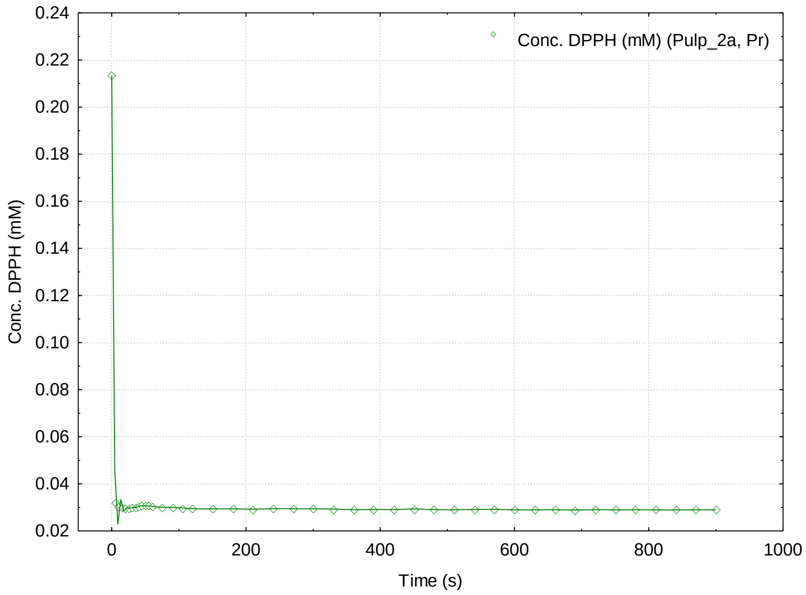

Figure S6. *DPPH· concentration* (mM) *versus Time* (s) for the ripe papaya pulp extract (code *Pr*), one determination.

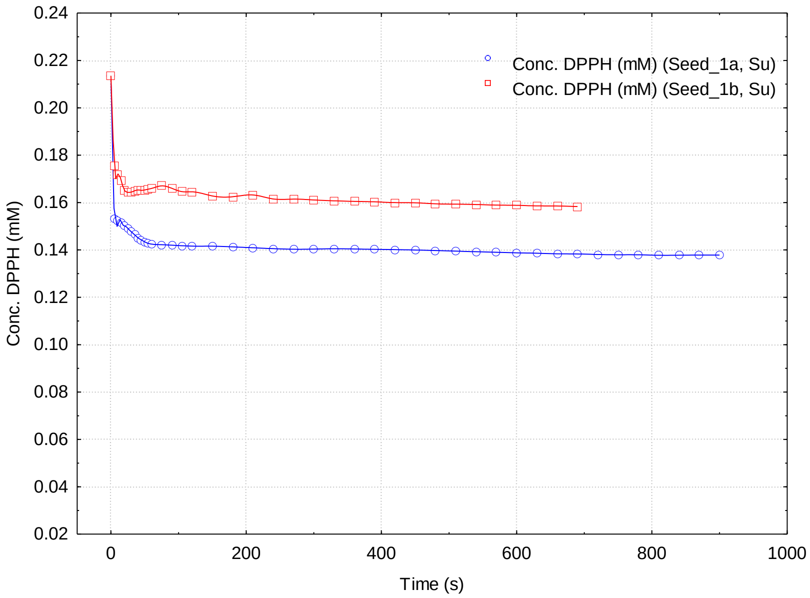

Figure S7. *DPPH· concentration* (mM) *versus Time* (s) for the unripe papaya seed extract (code *Su*), duplicate analysis.

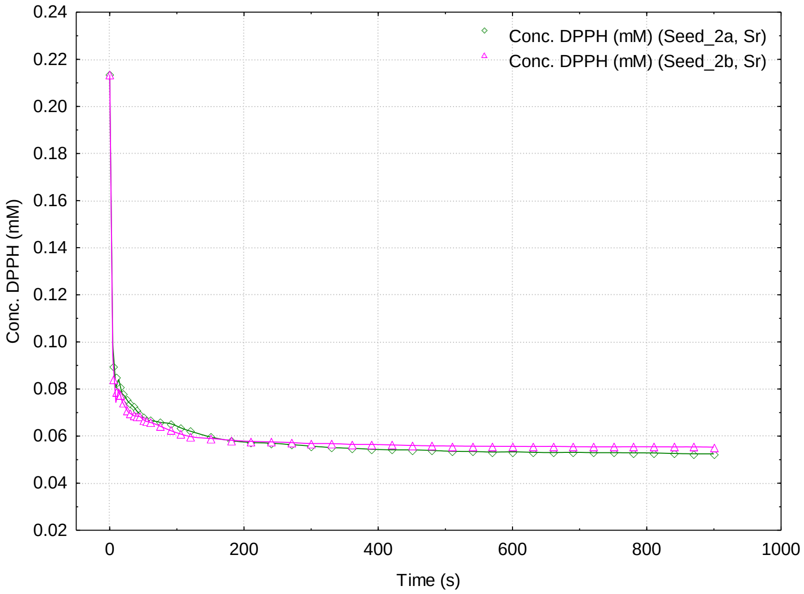

Figure S8. *DPPH· concentration* (mM) *versus Time* (s) for the ripe papaya seed extract (code *Sr*), duplicate analysis.

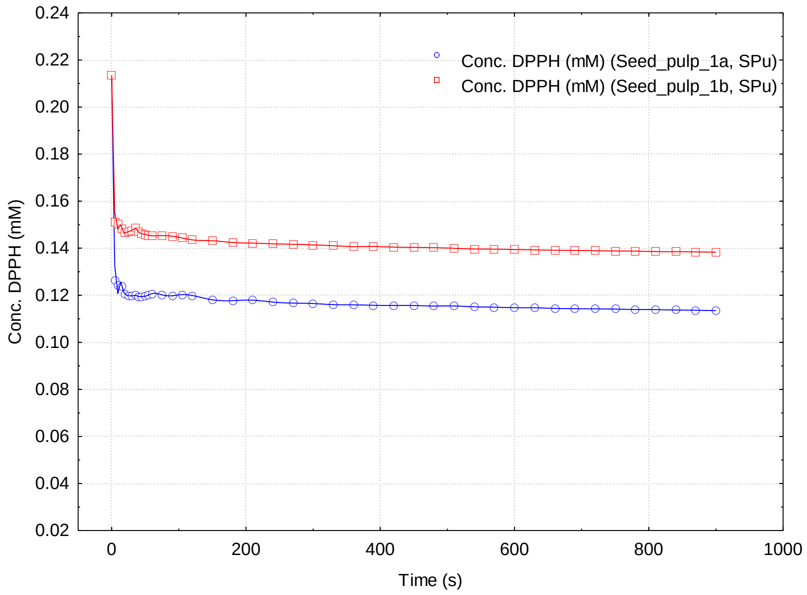

Figure S9. *DPPH· concentration* (mM) *versus Time* (s) for the unripe papaya seed-pulp extract (code *SPu*), duplicate analysis.

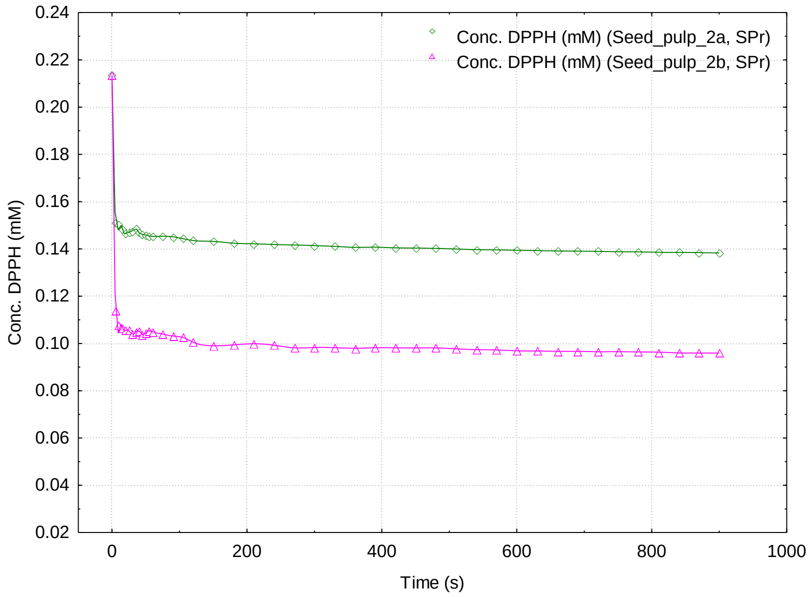

Figure S10. *DPPH· concentration* (mM) *versus Time* (s) for the ripe papaya seed-pulp extract (code *SPr*), duplicate analysis.

Table S8. Significant *p*-levels from the Fisher's LSD test (least significant difference) for the DPPH· reaction rate on the t_1 = *0-30 s* time range in the presence of papaya extracts (v_1, μM/s; unripe papaya peel, pulp, seed and seed-pulp: *Lu*, *Pu*, *Su* and *SPu*; ripe papaya peel, pulp, seed and seed-pulp: *Lr*, *Pr*, *Sr* and *SPr*;). *p*-level values lower than 0.05 are bolded.

	Lu	***Lr***	***Pu***	***Pr***	***Su***	***Sr***	***SPu***	***SPr***
Lu		**0.042626**	0.324667	**0.010951**	0.496345	**0.017784**	0.496345	**0.039135**
Lr			0.090636	0.088233	0.107066	0.309577	0.107066	0.920907
Pu				**0.015394**	0.808476	**0.027721**	0.808476	0.080651
Pr					**0.020171**	0.265136	**0.020171**	0.097144
Su						**0.039135**	1.000000	0.097144
Sr							**0.039135**	0.350108
SPu								0.097144
SPr								

Table S9. Significant *p*-levels from the Fisher's LSD test (least significant difference) for the DPPH· reaction rate on the t_1 = *0-30 s* time range in the presence of papaya extracts (v_1, μM/s; unripe papaya peel, pulp, seed and seed-pulp: *Lu*, *Pu*, *Su* and *SPu*; ripe papaya peel, pulp, seed and seed-pulp: *Lr*, *Pr*, *Sr* and *SPr*;). *p*-level values lower than 0.05 are bolded.

	Lu	***Lr***	***Pu***	***Pr***	***Su***	***Sr***	***SPu***	***SPr***
Lu		0.566691	0.445552	0.497436	0.723043	0.836951	0.639584	0.439860
Lr			0.146409	0.211887	0.347799	0.360103	0.293696	0.144029
Pu				0.988407	0.705437	0.486206	0.806830	0.989432
Pr					0.733519	0.556309	0.822418	0.997036
Su						0.836951	0.906179	0.697659
Sr							0.733940	0.478746
SPu								0.798573
SPr								

Table S10. Significant *p*-levels from the Fisher's LSD test (least significant difference) for the DPPH· reaction rate on the t_3 = *80-900 s* time range in the presence of papaya extracts (v_3, μM/s; unripe papaya peel, pulp, seed and seed-pulp: *Lu*, *Pu*, *Su* and *SPu*; ripe papaya peel, pulp, seed and seed-pulp: *Lr*, *Pr*, *Sr* and *SPr*;). *p*-level values lower than 0.05 are bolded.

	Lu	***Lr***	***Pu***	***Pr***	***Su***	***Sr***	***SPu***	***SPr***
Lu		**0.005997**	**0.003771**	**0.002367**	**0.003273**	**0.002592**	**0.003869**	**0.001733**
Lr			0.424297	0.060263	0.125043	0.168457	0.182084	0.059586
Pu				0.134699	0.293031	0.473669	0.425173	0.160836
Pr					0.597528	0.287532	0.442219	0.664336
Su						0.602578	0.793829	0.855970
Sr							0.820739	0.405762
SPu								0.633005
SPr								

Table S11. DPPH· reaction rates ($v_{1\text{-}3}$ – μM/s, at $t_{1\text{-}3}$ time ranges: 0-60 s, 60-180 s, and 180-900 s, respectively) for standard antioxidant compounds (*PG_1mM/0.2mM/0.1mM/0.05mM* – propyl gallate ethanolic solutions at concentrations of 1, 0.2, 0.1 and 0.05 mM; *CA_1mM/0.2mM/0.1mM/0.05mM* – caffeic acid ethanolic solutions at concentrations of 1, 0.2, 0.1 and 0.05 mM; *BHA_1mM/0.2mM/0.1mM/0.05mM* – *tert*-butylated hydroxyanisole ethanolic solutions at concentrations of 1, 0.2, 0.1 and 0.05 mM). Values are means of duplicate analysis, with *RSD* < 5% (from [1], with permission).

Nº	Code	DPPH· reaction rate on t_1 time range, v_1 (μM/s)	DPPH· reaction rate on t_2 time range, v_2 (μM/s)	DPPH· reaction rate on t_3 time range, v_3 (μM/s)
1	*PG_1mM*	1.90	-	0.001
2	*PG_0.2mM*	1.90	0.10	0.030
3	*PG_0.1mM*	1.20	0.05	0.015
4	*PG_0.05mM*	1.00	0.10	0.008
5	*CA_1mM*	2.50	0.40	0.023
6	*CA_0.2mM*	1.30	0.20	0.025
7	*CA_0.1mM*	1.30	0.07	0.007
8	*CA_0.05mM*	0.50	0.02	0.003
9	*BHA_1mM*	1.20	0.90	0.097
10	*BHA_0.2mM*	-	0.30	0.065
11	*BHA_0.1mM*	0.20	0.07	0.037
12	*BHA_0.05mM*	0.30	0.06	0.014

Table S12. DPPH· reaction rates ($v_{1\text{-}3}$ – μM/s, at $t_{1\text{-}3}$ time ranges: 0-30 s, 30-90 s, and 90-900 s, respectively) for other fruit extracts (*Pg_J(1:50/1:100)* – pomegranate raw juice, 1:50 and 1:100 dilution; *Pg_Rp/Wp(1:50/1:100)* – pomegranate red/white peel ethanolic extracts, 1:50 and 1:100 dilution; *Pg_P/S* – pomegranate pulp and seed ethanolic extracts, 1:50 and 1:100 dilution). Values are expressed as mean (±standard deviation, SD) for multiplicate analysis (n = 7 for *Pg_J* samples and n = 3 for *Pg_Rp*, *Pg_Wp*, *Pg_P* and *Pg_S* samples) (from [2], with permission).

Nº	Code	DPPH· reaction rate on t_1 time range, v_1 (μM/s)	DPPH· reaction rate on t_2 time range, v_2 (μM/s)	DPPH· reaction rate on t_3 time range, v_3 (μM/s)
1	*Pg_J(1:50)*	0.69 (±0.25)	0.26 (±0.13)	0.05 (±0.01)
2	*Pg_J(1:100)*	0.36 (±0.13)	0.14 (±0.06)	0.03 (±0.01)
3	*Pg_Rp(1:50)*	2.97 (±0.25)	0.23 (±0.12)	0.005 (±0.001)
4	*Pg_Rp(1:100)*	3.03 (±0.06)	0.47 (±0.23)	0.006 (±0.001)
5	*Pg_Wp(1:50)*	2.83 (±0.32)	0.30 (±0.00)	0.003 (±0.000)
6	*Pg_Wp(1:100)*	2.80 (±0.52)	0.60 (±0.00)	0.007 (±0.004)
7	*Pg_P(1:50)*	2.93 (±0.15)	0.37 (±0.06)	0.004 (±0.001)
8	*Pg_P(1:100)*	2.43 (±0.38)	0.60 (±0.10)	0.019 (±0.006)
9	*Pg_S(1:50)*	0.20 (±0.00)	0.07 (±0.02)	0.01 (±0.001)
10	*Pg_S(1:100)*	0.17 (±0.06)	0.05 (±0.02)	0.007 (±0.002)

References

1. Ivanovici, M.; Sicoe, G.; Hădărugă, D.I. Kinetics and antiradical activity of natural and synthetic phenolic compounds by DPPH method: a comparative study. *Journal of Agroalimentary Processes and Technologies* **2018**, *24*, 97-103.

2. Sicoe, G.; Oprinescu, C.I.; Golea, G.M.; Riviş, A.; Hădărugă, N.G. Kinetics on the DPPH· reaction with hydroalcoholic extracts from various pomegranate parts. *Journal of Agroalimentary Processes and Technologies* **2017**, *23*, 271-280.

3. Hădărugă, D.I.; Pantea, C.; Hădărugă, N.G. Antioxidant activity and kinetics on kiwi fruit (*Actinidia deliciosa*) ethanolic extracts by 2,2-diphenyl-1-picrylhydrazyl (DPPH·) method. *Journal of Agroalimentary Processes and Technologies* **2016**, *22*, 207-211.
